# An Updated Review on the Secondary Metabolites and Biological Activities of *Aspergillus ruber* and *Aspergillus flavus* and Exploring the Cytotoxic Potential of Their Isolated Compounds Using Virtual Screening

**DOI:** 10.1155/2021/8860784

**Published:** 2021-01-31

**Authors:** Fadia S. Youssef, Abdel Nasser B. Singab

**Affiliations:** ^1^Department of Pharmacognosy, Faculty of Pharmacy, Ain-Shams University, Cairo 11566, Egypt; ^2^Center for Drug Discovery Research and Development, Faculty of Pharmacy, Ain-Shams University, Cairo 11566, Egypt

## Abstract

The secondary metabolites and biological activities of *Aspergillus ruber* and *Aspergillus flavus* were comprehensively reported. About 70 compounds were isolated from both species that belong to different classes using conventional and advanced chromatographic techniques and unambiguously elucidated employing one- and two-dimensional nuclear magnetic resonance (1D and 2D NMR) and high resolution mass spectrometry (HRMS). Some of them displayed promising antiviral, anti-inflammatory, and antioxidant activities. *In silico* studies were conducted on human cyclin-dependent kinase 2 (CDK-2), human DNA topoisomerase II (TOP-2), and matrix metalloprotinase 13 (MMP-13) in an effort to explore the cytotoxic potential of the diverse compounds obtained from both *Aspergillus* species. 1,6,8-Trihydroxy-4-benzoyloxy-3-methylanthraquinone (23) revealed the most firm fitting with the active pockets of CDK-2 and MMP-13; meanwhile, variecolorin H alkaloid (14) showed the highest fitting within TOP-2 with ∆G equals to −36.51 kcal/mole. Thus, fungal metabolites could offer new drug entities for combating cancer. Relevant data about both *Aspergillus* species up to August 2020 were gathered from various databases comprising Scifinder (https://scifinder.cas.org/scifinder/login) for secondary metabolite-related studies; meanwhile, for biology-related articles, data were collected from both PubMed (http://www.ncbi.nlm.nih.gov/pubmed/) and Web of Knowledge (http://www.webofknowledge.com) as well.

## 1. Introduction

Fungi have been recently considered as a promising source of secondary metabolites that elicited a wide range of beneficial values both on the therapeutic and commercial scales. Recently, fungal metabolites have gained a great attention as an everlasting source of precious compounds that can serve as novel entities for various therapeutic approaches [1]. These metabolites belong to a vast array of chemical classes represented mainly by terpenoids, alkaloids, peptides, lactones, and steroids. Meanwhile, to these metabolites, various biological activities were assigned as anticancer, antiviral, antibacterial, and anti-inflammatory activities [2]. Fungi possess the advantage that they can be effectively cultured giving a high rate of reproduction with concomitant production of active metabolites [3]. Besides, many fungal metabolites showed suitable oral-bioavailability and appropriate physico-chemical characters being safer than synthetic moieties that are critical in the formulation of different dosage forms [1, 4].

Although, a large number of pharmaceutical products such as penicillins, griseofulvin, fucidin and ergot containing pharmaceutical products are of fungal origin but studies performed on fungal metabolites are still quite small [5, 6].

Fungal metabolites are usually isolated from the fungal culture medium undergoing fermentation followed by its extraction employing various solvents and its subsequent evaporation under vacuum at 40°C. The obtained extract is subjected to different conventional and advanced chromatographic techniques for the isolation of metabolites [7]. Meanwhile, the isolated fungal metabolites are structurally elucidated using 1D and 2D NMR (one- and two-dimensional nuclear magnetic resonance) and MS (mass spectrometry). The absolute configurations were further confirmed via Marfey's reactions in addition to Mosher's reaction and other chemical structural modification procedures [8].

Genus *Aspergillus* is a highly popular fungus which includes many species from which many metabolites belonging to different classes such as alkaloids, steroids, polyketides, peptides, and terpenoids were isolated. Some of these metabolites showed outstanding biological activities, particularly anticancer and antimicrobial [9, 10]. In this review, the secondary metabolites and biological activities of two *Aspergillus* species, namely, *Aspergillus ruber* and *Aspergillus flavus,* were comprehensively reported. Data were collected in an effort to give a full picture about the chemistry and biology of these two species that undoubtedly could help other researchers who wish to undergo further studies on these two reported species. The data compiled in this review were collected from various databases including PubMed (http://www.ncbi.nlm.nih.gov/pubmed), Web of Knowledge (http://www.webofknowledge.com), and SciFinder (https://scifinder.cas.org/scifinder/login)Additionally, in silico virtual studies were conducted on critical enzymes involved in the formation, progression, and metastasis of cancer as well, namely, human cyclin-dependent kinase 2 (CDK-2), human DNA topoisomerase II (TOP-2), and matrix metalloprotinase 13 (MMP-13) in an effort to explore the cytotoxic potential of both *Aspergillus* species isolated compounds as future perspectives.

## 2. Biological Activity and Secondary Metabolites Obtained from *Aspergillus ruber*

*A. ruber* is a fungus that is popular by possessing a substantial amount of secondary metabolites belonging to various classes in which some of them showed promising biological activities. Six compounds (1–6) were isolated from *A. ruber* using various chromatographic techniques and then structurally elucidated by comparing their 1D and 2D NMR and MS with that previously reported in literature. These compounds were determined to be echinulin (1), neoechinulin A (2), erythroglaucin (3), physcion (4), flavoglaucin (5), and isodihydroauroglaucin (6). The absolute configurations of (1) and (2) were further confirmed by advanced Marfey's method in which alanine was assigned to be L-Ala. Compounds (5) and (6) were assessed for antiviral potential versus human cytomegalovirus (HCMV) and herpes simplex-1 virus (HSV-1). Both compounds displayed a promising significant antiviral potential versus HSV-1 virus displaying EC50 values of 6.95 and 4.73 *μ*M, respectively, with moderate cytotoxic effect versus Vero cell. On the contrary, they showed no activity versus HCMV, in addition compound (6) revealed weak antibacterial effect [11]. Additionally, tetrahydroauroglaucin (7) was isolated in an abundant amount and low price from the fermentation culture media of the marine derived fungus, *A. ruber*. It showed a notable antibacterial activity [12, 13].

Besides, the cultivation of *A. ruber* obtained from crinoid on two different culture media, namely, rice solid and soybean, resulted in the variation in the obtained secondary metabolites as evidenced by the HPLC profiles of their extracts. This was followed by subsequent fractionation and purification using plethora of chromatographic techniques followed by characterization using various NMR and MS techniques that resulted in the identification of different secondary metabolites. From the soyabean extract, a new alkaloid compound, epoxyisoechinulin A (8) and fifteen known compounds (2, 9-22) were isolated. Meanwhile from the solid rice culture media, compounds (17, 4, 28) in addition to compounds (4, 22-29) were identified. The known isolated compounds are preechinulin (9), cyclo(Trp-Ana) (10), questinol (11), neoechinulin A (2), neochinulin *E* (12), neochinulin B (13), variecolorin H (14), variecolorin *J* (15), cryptoechinuline *G* (16), dihydroxyisoechinulin A (17), 2-(1,1-dimethyl-2-propen1-yl)-1H-indole-3-carboxaldenhyde (18), rubrocristin (19), neoechinulin *E* (20), eurotinone (21), physcion (4), asperflavin (22), 1,6,8-trihydroxy-4-benzoyloxy-3-methylanthraquinone (23), 2-methyleurotinone (24), 2-hydroxydiplopterol (25), catenarin (26), 2-*O*-methyl-9-dehydroxyeurotinone (27), and isodihydroauroglaucin (28). By comparing the metabolites obtained from both culture media, it was clearly obvious that the amount of alkaloids is higher in soybean culture medium due to its richness with nitrogen. Meanwhile, anthraquinone and polyketides are more abundant in rice culture medium that is rich in carbon, suggesting the induction of PKS (polyketide synthase) biosynthetic pathways triggered by the culture condition. However, testing the isolated compounds against a panel of microorganisms, namely, *Staphylococcus aureus*, *Vibrio alginolyticus*, *Exiguobacterium aurantiacum*, *Vibrio cholera*, *Escherichia coli*, *Vibrio parahaemolyticus*, *Salmonella*, *Shigella castellani*, *Vibrio vulnificus*, *Bacillus cereus*, *Morganella morganii,* and *Citrobacter freundii,* unfortunately showed no antibacterial activity against the tested strains [14–16]. Asperinines A (29) and B (30) are two new compounds isolated from *A. ruber* possessing tetrahydroanthrene and 1,4-anthraquinone moiety [17].

In addition, a red pigment, namely, erythroglaucin (31), was isolated from *A. ruber* through various chromatographic techniques, namely, thin layer chromatography and column chromatograph*y.* Upon reaction with ferrous ions (Fe^2+^), this pigment resulted in the formation of a dark blue complex that is found to be insoluble in chloroform, ether, methanol, water, and dimethylsulfoxide [18]. Moreover, from the ether extract of *A. ruber* culture media, a yellow pigment was isolated, purified, and characterized to be physcion (4) that is soluble in chloroform but insoluble in methanol. It also possess iron chelating properties as it can react with iron forming a reddish-brown colored complex postulating the probability that physcion may contribute to iron metabolism or transportation via fungal cells [19].

Additionally, three compounds that are diketopiperazines possessing dehydrotryptophan moieties, namely, isoechinulins A (32), B (33), and C (34), were isolated from *A. ruber;* their structures were unambiguously determined using ^13^C-NMR spectral data, taking into consideration the chemical shifts and the multiplicities as well. It is noteworthy to mention that isoechinulin A (32) is a potent inhibitor to the growth of silkworm larvae [20, 21]. Besides, compounds (35-36), two new compounds possessing indole moiety, were isolated from the same *Aspergillus species* and also displayed a potent inhibition to silkworm larvae growth [15, 16, 22].

**Figure 1:**
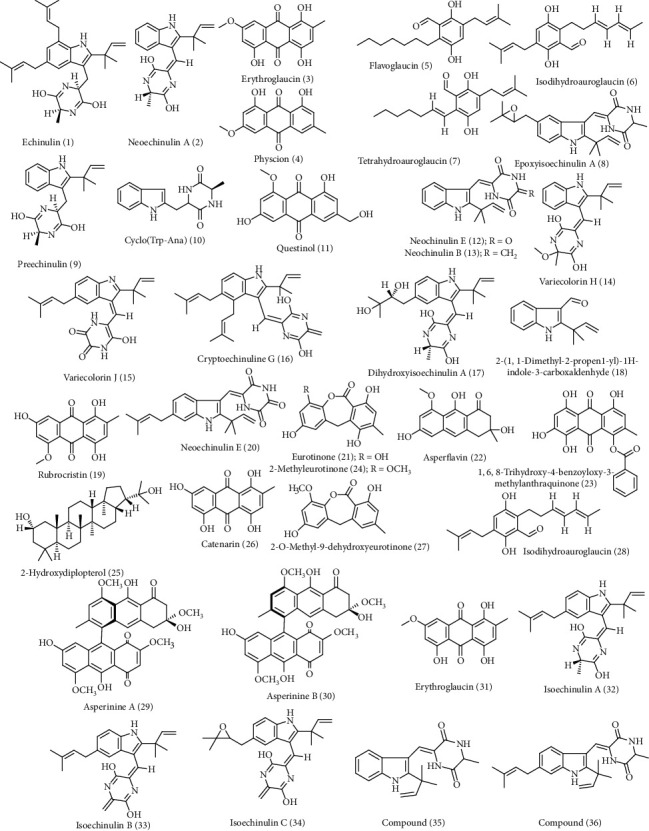
Secondary metabolites isolated from *Aspergillus ruber*.

Furthermore, tannase enzyme was effectively produced in high yield from *A. ruber* upon culturing on solid state fermentation medium [23]. It is noteworthy to highlight that tannase is an enzyme that effectively catalyzes the deesterification of tannins to glucose and gallic acid. This enzyme is of great importance for plant biomass recycling and in treatment of tannery effluents as well in addition to its beneficial value in the production of gallic acid that is of great pharmaceutical importance (Figure 1 displays secondary metabolites isolated from *A. ruber*).

## 3. Biological Activity and Secondary Metabolites Obtained from *Aspergillus flavus*

*A. flavus* is a well-known saprophyte and opportunistic pathogen as well that resulted in the production of multiple secondary metabolites [24]. *A. flavus* was found to be the highest productive strain of kojic acid (37) that is highly produced by an amount estimated by 18.61 g/L in a three-liter batch reactor and this production is greatly enhanced, employing the strategy of double pH. It is noteworthy to mention that kojic acid is highly popular in pharmaceutical and cosmetic preparation as a promising whitening agent for the skin [25]. In addition, two new compounds, namely, 5-acetoxy-3-hydroxy-3-methylpentanoic acid (38) and 5-chloro-2-methoxy-N-phenylbenzamide (39), were isolated from *A. flavus* in addition to other known compounds which are kojic acid methyl ether (40), cyclo(leucylprolyl) (41), uracil (42), linoleic acid (43), and glycerol linoleate (44). All the isolated compounds showed no cytotoxic effect against (KB-3-1) that is a human cervix carcinoma cell [26].

In a study carried on culture filtrate of *A. flavus,* it was found that the extract is rich in flavonoid and total phenolic contents estimated by 158.33 mg quercetin/mL and 65.77 mg GAE/mL of the crude extract, respectively. The crude extract displayed a potent antifungal and antibacterial activity against many common human pathogens. It also showed antioxidant activity manifested by its free radical scavenging behavior towards DPPH·(stable free diphenylpicrylhydrazyl radical) in which 700 *µ*g/mL of the extract scavenged 64.53% of the free radicals. Meanwhile, 2 mg/mL of the crude extract effectively inhibits RBCs hemolysis by 70% comparable to 78% inhibition elicited by ibuprofen, a standard drug [27].

Furthermore, *A. flavus* is a source of amino peptidases which have a plethora of commercial applications among which is their utilization to enhance the functional potential of protein products and develop flavor to cheese [28].

Moreover, cyclopiazonic acid, an indole-hydrindane-tetramic acid that acts as a neurotoxin, was produced by *A. flavus.* UHPLC triple-TOF HRMS was used to identify several CPA-type alkaloids from *A. flavus,* two of which were new, namely, 3-hydroxy-2-oxo CPA and 11,12-dehydro *α*-CPA; meanwhile eighteen compounds were identified from it which are *α*-CPA (45), *β*-CPA (46), *α*-CPA imine (47), cAATrp 2-oxo CPA (48), speradine A-D (49-52), 3-OH-speradine A (53), speradine F (54), speradine H (55), cyclopiamide A-F (56-61), and cyclopiamide *J* (62) [29].

**Figure 2:**
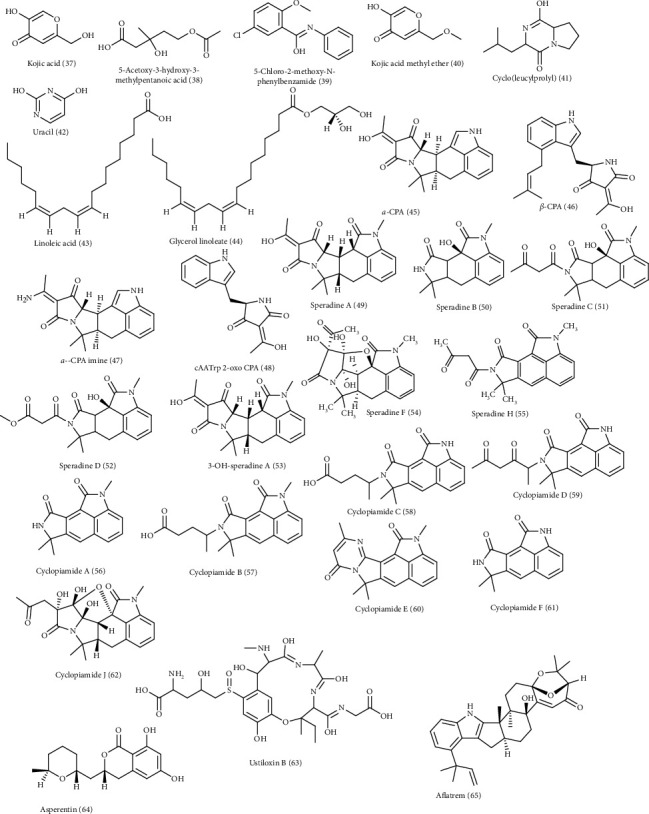
Secondary metabolites isolated from *Aspergillus flavus*.

**Figure 3:**
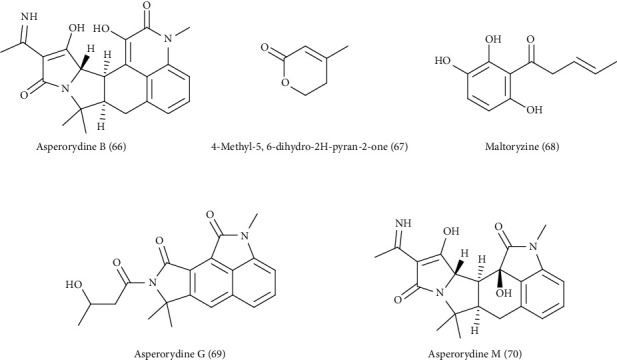
Secondary metabolites isolated from *Aspergillus flavus* (continued).

Ustiloxin B (63), a cyclic tetrapeptide compound, asperentin (64), and aflatrem (65), an indole diterpene, were also isolated from *A. flavus* [30–32]. It is noteworthy to mention that *A. flavus* is a rich source of aflatoxins mainly aflatoxin B1 that is considered to be an aggressive hepatocarcinogen in experimental models in addition to triggering of tumors in colon and kidneys. Aflatoxin B1 is changed to aflatoxin M1 that is equally carcinogenic [33].  Figure 2 illustrates the secondary metabolites isolated from *Aspergillus flavus*. Additionally, LC-MS analysis of the ethyl acetate extract of *A. flavus* associated with the soft coral *Sarcophyton ehrenbergi* led to the identification of seven compounds, namely, asperorydine B (66), 4-methyl-5,6-dihydro-2H-pyran-2-one (67), speradine A (53), maltoryzine (68), aflatoxine B1, asperorydine *G* (69), and asperorydine *M* (70) [34], (Figure 3).

The biological activities of both *A. ruber* and *A. flavus* are represented in Table 1.

## 4. Exploring the Cytotoxic Potential of Both *Aspergillus* Species Isolated Compounds Using Virtual Screening as Future Perspectives

Cancer is the biggest health problem facing the healthcare system worldwide. The major challenge appears from its diverse etiology, and its hazardous consequence is that it ultimately lead to death. Although many therapeutic regimes and protocols were developed, most of these treatments are only effective with about 40% of the case based on early diagnosis. Another challenge merged significantly that involved a dramatic exponential increase in the new cancer cases in the last decade, especially in the developing countries. In the Eastern Mediterranean Region (EMR), the increase in the expected cases reaches 1.8 folds in the next few years. Thus, cancer is considered the second cause of death in the developing countries and the fourth cause in the EMR [35].

Nowadays, management of cancer can be achieved mainly through different guidelines that involve surgery, radiation, and the use of chemotherapeutic agents. Furthermore, modifications of the known anticancer drugs to overcome multidrug resistance mechanisms proved to be inefficient in the majority of cases, and this potentiates the need to search for new safer lead drugs with lower side effects. Natural resources still represent the main focus for discovery of novel anticancer leading entities, whereas 60% of drugs used in its management are supplied from nature. Recently, marine sponges were proclaimed to be an excellent source of novel, effective entities displaying potent anticancer activity. Consequently, this encourages the investigation of related molecules. In spite of displaying a prominent activity in the experimental models, only few had passed to the clinical trials phase. Thus, there is an urgent demand for continual search for active compounds with neoteric nuclei [36].

Thus, molecular modelling studies were performed on crucial enzymes implicated in the formation, progression, and metastasis of cancer which are human cyclin-dependent kinase 2 (CDK-2) (PDB ID 1PXP, 2.30 Å), human DNA topoisomerase II (TOP-2) (PDB ID 4G0U, 2.70 Å), and matrix metalloprotinase 13 (MMP-13) (PDB ID 1XUD, 1.8 Å). In silico studies were performed on the previously listed enzymes which were downloaded from protein data bank (http://www.pdb.org) using Discovery Studio 2.5 (Accelrys Inc., San Diego, CA, USA), adopting C-docker protocol. Free binding energies (∆G) were calculated as mentioned previously for the most stable docking poses [37–39].

The three enzymes chosen to test the probable cytotoxic potential of the identified enzymes are human cyclin-dependent kinase, human DNA topoisomerase II, and matrix metalloproteinases (MMPs). Human cyclin-dependent kinases constitute enzyme collection that tremendously affects cell cycle occurrence and transcription. CDK2 firmly binds to cyclin A and *E* forming a complex with the latter that involved in the G1- to S-phase transition while its complex with the former eventually causes cell cycle progression via the S to M phase. Therefore, CDK2 inhibition resulted in an effective cell proliferation inhibition and consequently arrested cancer progression [40]. However, human DNA topoisomerase II adjusts the critical functions within the cells by causing massive changes with respect to the shape of alteration regarding the chromosomal DNA structure resulting in DNA unwinding affecting cell survival [41]. Regarding matrix metalloproteinases (MMPs), they are group of enzymes that are able to decompose extracellular matrix of vital components that promptly leads to cancer cell growth and metastasis and thus their inhibition constitutes an advanced strategy in combating cancer [42].

Data obtained from virtual screening of the identified compounds in the active pockets of human cyclin-dependent kinase 2 (CDK-2), human DNA topoisomerase II (TOP-2), and matrix metalloprotinase 13 (MMP-13) revealed that some of the docked compounds showed considerable binding affinities towards the tested proteins; however, others showed weak interactions manifested by the positive values of ∆G. Meanwhile 1,6,8-trihydroxy-4-benzoyloxy-3-methylanthraquinone (23) revealed the most firm fitting with the active pockets of both CDK-2 and MMP-13 displaying free binding energies of −47.41 and −37.81 kcal/mole, respectively. It showed superior binding when compared to Doxorubicin, the potent standard anticancer agent and to CK8 (CDK-2 inhibitor) but moderate activity when compared to PB4 (MMP-13 inhibitor). However, variecolorin H (14) showed the highest fitting score within the active center of TOP-2 with ∆G equal to −36.51 kcal/mole exceeding that of doxorubicin that showed −15.98 kcal/mole as free binding energy (Table 2).

**Figure 4:**
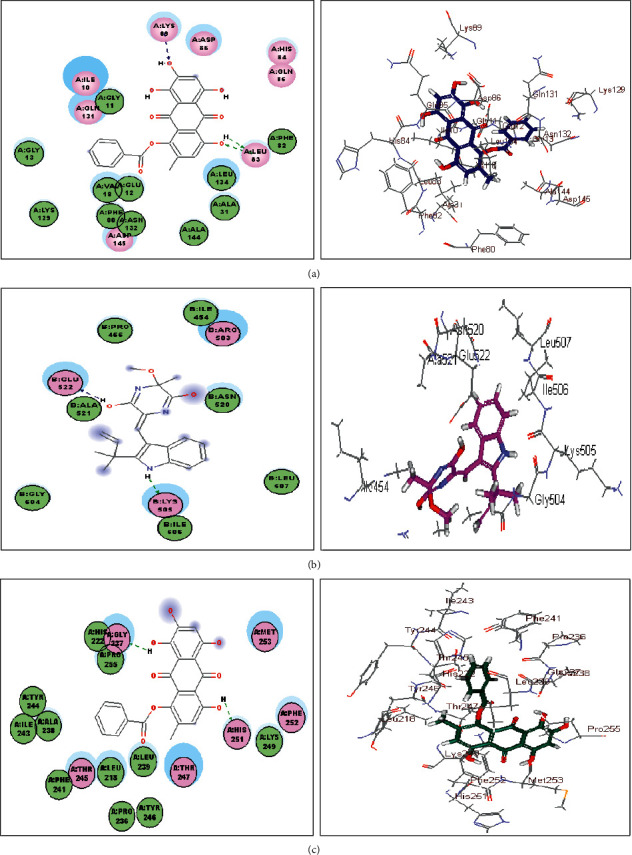
2D and 3D binding mode of 1,6,8-trihydroxy-4-benzoyloxy-3-methylanthraquinone (23) in the active center of human cyclin-dependent kinase 2 (CDK-2) (a), variecolorin H (14) in the active center of DNA topoisomerase II (TOP-2) (b), and 1,6,8-trihydroxy-4-benzoyloxy-3 methylanthraquinone (23) in the active center of matrix metalloprotinase 13 (MMP-13) (c) active centers using C-docker protocol.

The highest fitting of 1,6,8-trihydroxy-4-benzoyloxy-3-methylanthraquinone (23) could be explained by the virtue of formation of multiple bonds with the amino acid residues present at the active site represented by the formation of three H- bonds with Leu 83 and Lys 89 at CDK-2 active center and two H- bonds with Gly 237 and His 251 at MMP-13 active pocket. Meanwhile, variecolorin H (14) formed two H- bonds with Glu 522 and Lys 505 at TOP-2 active site as revealed in Figure 4.

## 5. Conclusion

In this study, it was concluded that nearly about seventy secondary metabolites were isolated from two *Aspergillus* species, namely, *Aspergillus ruber* and *Aspergillus flavus*. They were unambiguously elucidated employing one- and two-dimensional nuclear magnetic resonance (1D and 2D NMR) in addition to high resolution mass spectrometry (HRMS). Some of them displayed promising anticancer, antiviral, and antimicrobial activities; meanwhile, the others displayed no activity that necessitates further investigation. In silico studies judged by different proteins, inhibition revealed that some of the identified compounds showed considerable cytotoxic potential with 1,6,8-trihydroxy-4-benzoyloxy-3-methylanthraquinone and variecolorin H exhibited the highest activity. Further in vitro followed by in vivo studies should be conducted to confirm the in silico studies. Thus, more highlights should be shed on the discovery of new drug entities combating cancer and other debilitating disorders derived from fungal metabolites.

## Figures and Tables

**Table 1:** The biological activities of both *A. ruber* and *A. flavus*.

Compound	Genus	Biological activity	References
Flavoglaucin (5)	*A. ruber*	Promising significant antiviral potential versus HSV-1 virus displaying EC_50_ value of 6.95 *μ*M Moderate cytotoxic effect versus Vero cell	[10]
Isodihydroauroglaucin (6)	*A. ruber*	Promising significant antiviral potential versus HSV-1 virus displaying EC_50_ value of 4.73 *μ*M Moderate cytotoxic effect versus Vero cell Weak antibacterial effect	[10]
Tetrahydroauroglaucin (7)	*A. ruber*	Notable antibacterial activity	[11, 12]
Isoechinulin A (32)	*A. ruber*	Potent inhibition to the growth of silkworm larvae	[14, 15, 21]
Compounds (35-36)	*A. ruber*	Potent inhibition to the growth of silkworm larvae	[14, 15, 21]
Kojic acid (37)	*A. flavus*	Promising whitening agent for the skin	[24].
Tannase enzyme	*A. ruber*	Treatment of tannery effluents Production of gallic acid	[22]
Total extract	*A. flavus*	Potent antifungal and antibacterial activity against many common human pathogens. Promising antioxidant activity manifested by its free radical scavenging behavior towards DPPH· Effective inhibition of RBC hemolysis by 70% (at 2 mg/mL of the crude extract) comparable to 78% inhibition elicited by ibuprofen	[26]
Amino peptidases	*A. flavus*	Functional potential of protein products and develop flavor to cheese	[27]

**Table 2:** Free binding energies (∆G) in kcal/mol for compounds identified from *A. ruber* and *A. flavus* in human cyclin-dependent kinase 2 (CDK-2), DNA topoisomerase II (TOP-2), and matrix metalloprotinase 13 (MMP-13) active centers using C-docker protocol.

Compound	CDK-2	TOP-2	MMP-13
Echinulin (1)	14.07	26.35	FD
Neoechinulin A (2)	−22.96	−5.07	−26.00
Erythroglaucin (3)	−37.21	−19.45	−35.68
Physcion (4)	−35.63	−16.23	−32.68
Flavoglaucin (5)	−21.40	−3.53	−25.23
Isodihydroauroglaucin (6)	−0.82	15.03	−2.97
Tetrahydroauroglaucin (7)	−8.02	8.27	−15.45
Epoxyisoechinulin A (8)	−22.39	−2.13	−8.94
Preechinulin (9)	−21.94	−10.72	−20.062
Cyclo(Trp-Ana) (10)	−26.50	−14.94	−29.04
Questinol (11)	−37.08	−19.77	−33.94
Neochinulin *E* (12)	−17.39	−7.88	−18.84
Neochinulin B (13)	−21.03	−4.82	−22.88
Variecolorin H (14)	−16.99	−36.51	−24.82
Variecolorin *J* (15)	FD	36.51	29.86
Cryptoechinuline *G* (16)	15.29	30.36	FD
Dihydroxyisoechinulin A (17)	−25.96	−5.31	−8.262
2-(1_1-Dimethyl-2-propen1-yl)-1H-indole-3-carboxaldenhyde (18)	−15.05	−5.94	−19.58
Rubrocristin (19)	−36.00	−17.65	−30.94
Neoechinulin *E* (20)	−5.90	11.03	−7.58
Eurotinone (21)	−23.47	−11.828	−25.39
Asperflavin (22)	−17.88	−5.264	−14.27
1,6,8-Trihydroxy-4-benzoyloxy-3-methylanthraquinone (23)	−47.41	−23.38	−37.81
2-Methyleurotinone (24)	−20.48	−7.72	−19.16
2-Hydroxydiplopterol (25)	94.27	80.51	FD
Catenarin (26)	−5.05	−5.07	−4.66
2-O-Methyl-9-dehydroxyeurotinone (27)	−18.19	−7.014	−21.30
Isodihydroauroglaucin (28)	−0.82	15.02	−2.97
Asperinine A (29)	FD	22.17	FD
Asperinine B (30)	FD	26.36	FD
Erythroglaucin (31)	−37.21	−19.45	−35.68
Isoechinulin A (32)	−1.36	14.23	11.73
Isoechinulin B (33)	−8.89	14.26	1.85
Isoechinulin C (34)	−26.94	−3.48	−6.28
Compound (35)	−20.47	−3.81	−21.91
Compound (36)	−5.61	15.21	−1.61
Kojic acid (37)	−16.53	−10.71	−16.37
5-Acetoxy-3-hydroxy-3-methylpentanoic acid (38)	−25.67	−19.78	−29.52
5-Chloro-2-methoxy-N-phenylbenzamide (39)	−28.82	−11.4486	−32.0189
Kojic acid methyl ether (40)	−16.99	−10.3046	−18.7795
Cyclo(leucylprolyl) (41)	−13.27	−2.68683	−18.1432
Uracil (42)	−23.44	33.38	−24.07
Linoleic acid (43)	−20.39	−0.25	−23.88
Glycerol linoleate (44)	−21.40	1.915	−25.87
*α*-CPA (45)	18.63	30.26	45.24
*β*-CPA (46)	6.77	25.60	4.12
*α*-CPA imine (47)	22.87	33.67	39.92
cAATrp 2-oxo CPA (48)	−10.64	2.20	−15.32
Speradine B (50)	6.99	16.18	9.94
Speradine C (51)	−5.16	13.21	15.64
Speradine *D* (52)	−4.62	13.76	16.68
3-OH-speradine A (53)	47.50	49.92	FD
Speradine F (54)	70.29	48.89	FD
Speradine H (55)	17.00	35.30	26.63
Cyclopiamide A (56)	29.32	38.30	27.17
Cyclopiamide B (57)	14.91	34.73	20.46
Cyclopiamide C (58)	17.89	32.15	23.19
Cyclopiamide *D* (59)	20.78	31.61	21.79
Cyclopiamide *E* (60)	29.65	50.31	47.67
Cyclopiamide F (61)	30.32	40.30	29.17
Cyclopiamide *J* (62)	36.08	50.93	FD
Ustiloxin B (63)	−21.82	4.91	FD
Asperentin (64)	−25.95	−7.46	−25.53
Aflatrem (65)	75.33	84.32	FD
CK8 (CDK-2 inhibitor)	−39.34	ND	ND
Doxorubicin (TOP-2 inhibitor)	−39.99	−15.98	−16.29
PB4 (MMP-13 inhibitor)	ND	ND	−73.00

Positive values indicate unfavorable interaction. FD, fail to dock; ND, not done; CK8, N-[4-(2,4-dimethyl-thiazol-5-yl)-pyrimidin-2-yl]-N′, N′-dimethyl- benzene-1,4-diamine; PB4, N, N′-bis (4-fluoro-3-methylbenzyl) pyrimidine-4,6-dicarboxamid.
